# Beta-cyclodextrin inclusion complexes of citral and linalool inhibit *Escherichia coli* on cooked chicken: Focus on their synergistic antibacterial effects

**DOI:** 10.1016/j.fochx.2025.103248

**Published:** 2025-11-04

**Authors:** Wanying Zhu, Yuhe Dong, Tong Wu, Haohan Jing, Zikai Li, Xi Yu, Ying Xiao, Tian Zhong

**Affiliations:** a Faculty of Chinese Medicine, Macau University of Science and Technology, Macao SAR, China; b Faculty of Medicine, Macau University of Science and Technology, Macao SAR, China

**Keywords:** Essential oil, Plant-derived compounds, Antibacterial, Food preservation

## Abstract

Citral and linalool are two well-studied antibacterial essential oil compounds. However, their synergistic antibacterial effects have not been fully elucidated. This study explored their synergistic mechanism against *Escherichia coli* and the potential of their β-cyclodextrin (β-CD) inclusion complexes in food preservation. The combination of citral and linalool demonstrated enhanced efficiency in damaging bacterial cell membranes, inducing protein leakage, causing structural collapse, and inhibiting biofilm formation, reducing extracellular polymeric substance (EPS), and impairing bacterial motility compared to the individual use of either compound. Furthermore, the β-CD inclusion complexes of these two compounds exhibited synergistic effects in cooked chicken by inhibiting total viable counts (TVC) to 3.31 Log CFU/g after 3 days of storage, which is lower than the single-use groups (4.07 and 3.78 Log CFU/g for citral and linalool, respectively). These complexes also decreased total volatile basic nitrogen (TVB-N) accumulation and prevented pH increase, thereby enhancing food safety and quality.

## Highlights

• This work represents the first study on the synergistic effect of citral and linalool against *E. coli*.
• Synergistic mechanism disrupts membranes, increases ROS, and inhibits biofilm formation.
• The inclusion complex containing citral and linalool inhibits TVC in cooked chicken.
• The inclusion complex containing citral and linalool extends chicken's shelf-life.

## 1 Introduction

Food contamination by bacteria is becoming a growing global concern that will cause a series of health problems and great economic loss. *Escherichia coli* is frequently detected as the most prevalent Gram-negative bacteria in contaminated water and soil. Cooked meat products are commonly contaminated with *E. coli* during the production process (Keeratipibul et al., 2009). Because *E. coli* contamination cannot be easily identified through visual inspection, it frequently leads to foodborne illnesses and acute infections, posing a significant public health concern (Rani et al., 2021; Singha et al., 2023) Nowadays, chemical medicine and synthetic antibiotic are widely used on bacterial control. However, due to the rise and dissemination of muti-drug resistant bacteria, controlling bacterial infection has become increasingly challenging. Furthermore, synthetic antibiotic also contribute to environmental pollution (Calo et al., 2015). Therefore, an increasing number of studies have started to focus on investigating the antibacterial effects of natural products, especially essential oils. Essential oils may provide a natural and safe alternative for the treatment of bacterial infections with fewer side effects, non-toxic, and environmentally friendly compared to conventional antibacterial drugs (Li et al., 2022).

Essential oils typically consist of a blend of volatile compounds (Bakkali et al., 2008), and their overall antibacterial effect represents the collective activity of various chemical components within them (Ju et al., 2022). Varieties of combinations of individual essential oil compounds have shown synergistic antibacterial effects. A study conducted on fresh-cut cucumber reported that the mixture of cineole and carvacrol significantly reduced the *E. coli* population by increasing drug bioavailability, inhibiting the normal functions of nucleic acids and proteins, and regulating the transcription levels of virulence genes (Addo et al., 2023). Huang et al. (2021) reported that cinnamaldehyde and cinnamic acid exhibited a synergistic antibacterial effect against *Serovar pullorum* by interfering with membrane biosynthesis and suppressing bacterial cell proliferation. Additionally, the combination of citral and carvacrol has been proved to reduce infections caused by *Cronobacter sakazakii* in reconstituted infant formula through synergistic disruption of bacterial cell morphology and strong antibiofilm activity (Cao, Zhou, et al., 2021). In the essential oil industry, synthetic technology has gradually replaced traditional plant source extraction due to purity defects and process costs (Ghasemy-Piranloo et al., 2022). Therefore, understanding the composition of key antibacterial compounds in essential oils and their optimal proportions in practice is essential to guide and develop the synthetic essential oil industry.

However, compounds from essential oils generally not applied directly to food due to their low water solubility, active volatility and potential impact on flavor (Ju et al., 2019). Encapsulation is a technique that protects essential oils by the presence of one or more wall materials, which might minimize their direct contact with food components and boost antibacterial efficacy (Nair et al., 2022). Cyclodextrins (CDs) are cyclic carbohydrates derived from starch. Among the natural CDs, the most suitable CD has been β-CD, owning to its suitable size to encapsulate most of the essential oil compounds (Paiva-Santos et al., 2022). Its lipophilic chamber that allows the system to include lipophilic elements and their hydrophilic outer surface ensures effective complex dissolution in an aqueous environment (Ravindran Maniam et al., 2022). When forming an inclusion complex with essential oils, the guest molecule with a suitable size and shape is enclosed into the cavity within a solid structure formed by host molecules. Encapsulating essential oils with β-CD showed following advantages: 1) suppress volatility and increase stability of essential oil; 2) transform liquid compounds into crystalline form; 3) mask the unpleasant smell and taste; 4) avoid unexpected incompatibilities; 5) increase bioavailability (Marques, 2010) What is more, a substantial body of research has demonstrated that β-CD serves as an excellent packaging material for the encapsulation of essential oils used in food product preservation to antimicrobial (Ji et al., 2023).

Linalool, an acyclic monoterpene tertiary alcohol (monoterpenol), can be found in the essential oils of various plants that widely spread all around the world (Pereira et al., 2018). Citral, a monoterpene aldehyde and contains two isomers (geranial and neral), can mainly be found in citrus fruits and lemongrass (Saddiq & Khayyat, 2010). Studies have shown that both citral and linalool have antibacterial effects when applied individually. Linalool treatment can effectively inhibit the growth of *Shewanella putrefaciens* (Guo et al., 2021) and *Pseudomonas aeruginosa* (Liu et al., 2020) by destroying the structure and function of their cell walls and membranes. It can also restrain the growth of *Shigella sonnei* by increasing intracellular reactive oxygen species (ROS) levels, inducing oxidative stress and lipid peroxidation, and damaging cell membrane integrity (Su et al., 2022). Citral has a similar antibacterial mechanism as linalool. It is effective against *Yersinia enterocolitica* by changing the cellular morphology and intracellular soluble proteins, decreasing cell membrane integrity, and causing oxidative stress (Kang et al., 2022). Citral has also been demonstrated to suppress *Staphylococcus aureus* growth by damaging cell membrane and inhibiting metabolic activity and bacteria cell growth (Dai et al., 2023). Linalool has been reported to has the potential to significantly improve the overall antibacterial effectiveness of essential oils as an adjunct ingredient (Pereira et al., 2018). Moreover, a synergistic antifungal effect has been observed with the combination of citral and eugenol (Ju et al., 2020). Nevertheless, research on the combined antimicrobial effects of citral and linalool remains limited. To date, the only relevant study is that of Prakash and Vadivel (2020), who prepared emulsions containing citral and linalool to determine their minimum inhibitory concentration (MIC) against *Listeria monocytogenes*. However, no synergistic effect was observed in all the seven prepared nanoemulsions with different proportions.

**Fig. 1:**
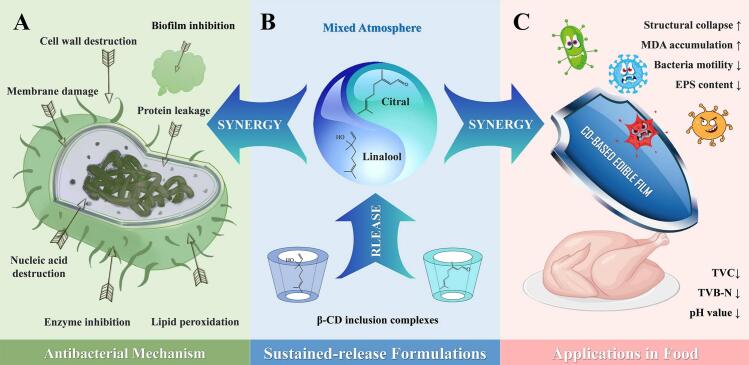
Schematic diagram of synergistic antibacterial mechanism of citral and linalool against *Escherichia coli* (A) and their β-cyclodextrin inclusion complexes (B) for extending the shelf-life of cooked chicken products (C).

Therefore, this study represents the first investigation into the synergistic mechanism of citral and linalool against *E. coli*, and verifies the antibacterial efficacy of their combined sustained-release formulation in a real food model. The potential mechanism of their synergistic antibacterial effect will be analyzed via extracellular protein leakage assays, cell morphological imaging, and malondialdehyde (MDA) measurements (Fig. 1A). Additionally, the synergistic inhibitory effects of this combination on *E. coli* biofilm formation will be evaluated through crystal violet (CV) staining, extracellular polymeric substances (EPS) production analysis, and swimming/swarming motility tests. Furthermore, β-CD inclusion complexes of these two compounds were prepared (Fig. 1B) and applied to cooked chicken meat. Changes in pH, total viable bacterial count (TVC), total volatile basic nitrogen (TVB-N), and color parameters during storage were assessed to validate their synergistic efficacy in extending the shelf-life of real food models (Fig. 1C).

## 2 Material and methods

### 2.1 Reagents and strains

Citral (mixture of α-citral and β-citral, 97 %, *w*/*v*), linalool (99 %, w/v), carvacrol (99 %, w/v), citronellal (96 %, w/v), cinnamaldehyde (98 %, w/v), thymol (98 %, w/v), vanillin (99 %, w/v), and eugenol (99 %, w/v), were purchased from Macklin Biochemical Technology Co., Ltd. Lysogeny broth (LB), Mueller-Hinton broth (MHB), nutrient broth (NB), nutrient agar (NA), potato dextrose agar (PDA), and potato dextrose water (PDW) were obtained from Qingdao Haibo Biological Technology Co., Ltd. All other reagents used in this study were of analytical grade. Before each experiment, *E. coli* strain (ATCC25922), *S. aureus* strain (CICC 21600), and *L.* *monocytogenes* strain (CICC 25021) was activated at 37 °C for 12 h with shaking. After activation, 200 μL of bacterial suspension was transferred into 20 mL fresh LB medium for 18 h culture (37 °C, 180 rpm) to achieve a bacterial population of ∼10^9^ CFU/mL. Prepared bacterial suspension was temporarily storage 4 °C for later use. For fungi, *Penicillium* spp. (isolated from naturally infected oranges) and *Aspergillus niger* (ATCC 16404) were inoculated onto PDA petri dishes and incubated at 28 °C for 3 days to ensure sufficient spores production. Spore suspensions were obtained by washing spores from the PDA plates with sterile PBS containing 1 % Tween-80.

### 2.2 Minimum inhibitory concentration (MIC)

The MIC of linalool, carvacrol, citronellal, cinnamaldehyde, citral, thymol, vanillin, and eugenol were determined using a double broth dilution method as described in previous studies but with some modifications (Addo et al., 2023; Ferreira et al., 2018). This test was carried out in Mueller-Hinton broth (MHB) with an initial population of around 10^6^ CFU/mL. Firstly, all the essential oil components were dissolved in 50 % ethanol, and 100 μL of MHB was pipetted into all wells of 96-well plate. Secondly, 100 μL of antibacterial solution was added to the first well of the rows. Thirdly, removed 100 μL mixture from the first well of each row, added it to the second well, mix it well and then repeat backwards. Next, 100 μL of the *E. coli* suspension (10^6^ CFU/mL) and 10 μL of 0.5 % 2,3,5-Triphenyltetrazolium chloride (TTC) solution were then pipetted into each well, resulting in three replicates of the same concentration of each essential oil compounds. Finally, the plate was cultured in an incubator at a constant temperature (37 °C) for 24 h, and the endpoint was determined visually by observing the presence of a red deposit, as described by de Souza et al. (2024). The samples containing equal amounts of ethanol without essential oil compounds were set as control group. The lowest concentration at which a well showed no bacterial growth was considered the MIC for that essential oil compounds.

The MIC values of citral and linalool against fungi were determined as described by Ju et al. (2020). In brief, the test was conducted in PDW, and the spore suspension was adjusted to a concentration of 10^5^ CFU/mL. The procedures performed on the 96-well microplate followed the same protocol as the previously described bacterial MIC determination method. After incubation at a constant temperature of 28 °C for 24 h, the results were visually assessed.

### 2.3 Fractional inhibitory concentration (FIC)

To determine the FIC index between linalool and seven other essential oil compounds, the checkerboard method was employed (Bassolé et al., 2010). The experiment dosage were set based on the study by (Cao, Zhou, et al., 2021). Bacterial cultures (1 × 10^6^ CFU/mL) and fungal spore suspensions (1 × 10^5^ CFU/mL) were added to each well of a 96-well microplate, respectively. The cell culture was treated with these two substances, which are combined in dual-axes (x and y) format and added to each well of the microplate to obtain final concentrations of 4 ×, 2 ×, 1 ×, 1/2, 1/4, 1/8, 1/16, and 1/32 MIC. A 0.5 % TTC solution was added to each well of the bacteria test plate as an indicator. Finally, the plates were incubated for 24 h at 37 °C. FICs were calculated as follows:

(1) $$\mathrm{FIC}=\frac{{\mathrm{MIC}}_{A\;\mathrm{in combination}}}{{\mathrm{MIC}}_{A\;\mathrm{alone}}}+\frac{{\mathrm{MIC}}_{B\;\mathrm{in combination}}}{{\mathrm{MIC}}_{B\;\mathrm{alone}}}$$

### 2.4 Viable bacterial counts

The bacterial inhibition affect was carried out in MHB. The prepared *E. coli* suspensions was diluted to a population of ∼10^6^ CFU/mL. The suspension was added in a 1.5 mL tube and mix with 95 % ethanol diluent of two essential oils to obtain the required final concentrations 1/2 MIC of individual citral (marked as 1/2C) and linalool (marked as 1/2L) and in combination (1/4C + 1/4L). The tubes were constant temperature incubated in 37 °C without shaking, then all samples were diluted and spread on LB agar plate after 24 h. The number of viable cells was determined by colony forming units (CFU) from each sample.

### 2.5 Extracellular protein leakage

The leakage of intracellular protein was quantitatively analyzed by BCA Protein Quantitation Kit (Nanjing Jiancheng, China). Briefly, after incubation at 37 °C for 24 h, the bacterial culture medium was centrifuged at 5000 rpm for 10 min to remove supernatant, washed by PBS thrice. Bacterial pellet was resuspended with sterile PBS to ∼10^6^ CFU/mL and incubated with individual citral and linalool (1/2C and 1/2L) and in combination (1/4C + 1/4L) at 37 °C. Following a 2-h incubation period, the samples were centrifuged at 5000 rpm for 10 min and collect the supernatant. The working solution was co-incubated with sample supernates at a ratio step by step according to the instructions of the BCA Protein Quantitation Kit. Setting blank tube by adding same amount of H_2_O into working solution. Finally, the content of released intracellular protein was analyzed by measuring the absorbance value of each sample at 562 nm by a microplate reader (Dai et al., 2023).

### 2.6 Scanning electron microscopy (SEM)

*E. coli* was adjusted to 10^8^ CFU/mL then co-incubate for 2 h with (1/2C, 1/2 L, 1/4C + 1/4L and 1/2C + 1/2L) and without (Control) antimicrobials. After washed with PBS twice, bacterial cell samples were eluded and fixed with 2.5 % v/v glutaraldehyde solution overnight and following with dehydrating in gradient ethanol solution (50–100 % v/v) for 10 min each as post-fixation. Finally, all fixed cells were air-dried and mounted on a SEM sample holder and coated with gold before visualization.

### 2.7 Malondialdehyde (MDA) content assay

*E. coli* was treated according to the description from Su et al. (2022) with proper modification. The overnight bacterial culture was first adjusted to the population of ∼10^8^ CFU/mL with LB broth and transferred into 1.5 mL tubes, mixed with citral and linalool to achieve preset experimental concentration (1C, 1L, 1/2C + 1/2L). All experiment tubes were vortex well mixed and placed in a 37 °C thermostatic incubator for 1 h. Then removed the incubate broth after centrifuge at 5000 rpm for 10 min. The change in MDA in *E. coli* was evaluated using the lipid peroxidation MDA test kit (Beijing Solarbio Science & Technology, Beijing, China). *E. coli* precipitates were re-suspended with MDA extraction solution then the mixtures were subsequently treated by ultrasonication in ice bath to help disrupting bacterial cell and release MDA. Following centrifugation (8000 ×*g*, 10 min, 4 °C), the supernatant was combined with the MDA working solution and then heated in boiled water bath for 1 h. Cell mass was removed by centrifuging at 1000 ×g for 10 min after ice bath cooled. MDA content was calculated by detecting the absorption of each supernatant sample at the wavelengths of 600 nm and 536 nm with microplate reader (FLUO star Omega, BMG Labtech, Germany).

### 2.8 Anti-biofilm formation activity

The experiment procedure was according to previous method with slight modification (Lim et al., 2022). An overnight grown culture was adjusted to ∼10^8^ CFU/mL in MH broth containing 1/2C, 1/2L and in combination 1/4C + 1/4L. The wells containing inoculum without essential oils served as growth controls. About 0.8 mL of culture was incubated for 48 h on a 24-well plate at 37 °C without shaking. Mature biofilm formed was washed twice with PBS to remove the loosely attached cells then stained with 0.1 % crystal violet (CV) for 15 min. Next, the microplates were washed three times with sterile distilled water to remove excess CV. The attached biofilm formed was quantified by solubilizing with 95 % ethanol and absorbance at 595 nm was read. All experiments were performed in triplicate, and the biofilm inhibitory efficacy was calculated using the following formula:

(2) $$\mathrm{Biofilm inhibition}\;\left(\%\right)=\frac{\mathrm{OD}595_{\left(\mathrm{control}\right)}-\mathrm{OD}595_{\left(\mathrm{sample}\right)}}{\mathrm{OD}595_{\left(\mathrm{control}\right)}}\times 100\%$$

### 2.9 EPS production

EPS production affected by essential oils was quantified by the phenol‑sulfuric acid method (Cao, Liu, et al., 2021). The overnight bacterial culture was inoculated into LB broth (1 × 10^8^ CFU/mL) supplemented with citral and linalool at different concentrations (1/2C, 1/2L, 1/4C + 1/4L) and incubated in a 24-well microplate for 48 h to form biofilms. The biofilm of all wells was resuspended to the culture medium and then were centrifuged at 5000 rpm for 15 min to remove bacterial cells. The supernatants were collected and mixed with 95 % (v/v) ethanol of 3-fold volume, respectively, followed by precipitated at 4 °C for 24 h. Afterwards, the precipitations, were gathered after centrifugation at 5000 rpm for 15 min. The total polysaccharide was subsequently mixed with water, phenol (5 %, v/v), and the concentrated sulfuric acid in the ratio of 1:1:5 (v:v:v) to carry out the reaction for 20 min. A 490 nm wavelength absorbance measurement was used to calculate the contents of EPS.

### 2.10 Swimming and swarming test

The motility tests were performed by using a slightly modified version of the prior procedure (Addo et al., 2023). The swarming motility was assessed on LB agar plates containing 1.5 % (w/v) agar and swimming motility was determined on LB plate with 0.3 % (w/v) agar. In brief, all testing plates were added essential oil ethanol solutions before agar solid to obtain various concentrations of 1/2C, 1/2L, 1/4C + 1/4L. Plate without essential oils was regarded as the control. Overnight culture of *E. coli* was adjusted to the concentration of ∼10^8^ CFU/mL and 2 μL of bacterial suspension was spotted at the center of both swimming and swarming test disks then incubated at 37 °C for 24 h. Finally, the motility inhibiting effect of each experiment groups were recorded by photograph.

### 2.11 Preparation of β-CD inclusion complexes

All inclusion complexes were produced utilizing the coprecipitation method, with slight modifications. β-CD (4 g) was dissolved in 100 mL of distilled water at 75 °C to obtain supersaturation aqueous solution. After β-CD solution were cooled to room temperature, 0.64 g of linalool or citral were added in severally, then the mixture was kept stirring for 4 h to acquire Citral@β-CD or Linalool@β-CD suspension. To obtain Citral@β-CD + Linalool@β-CD (CL@β-CD), equal volumes of Citral@β-CD and Linalool@β-CD suspensions were evenly mixed. All cyclodextrin inclusion suspensions were suction-filtrated, and the precipitate was collected then dried at 30 °C.

### 2.12 Entrapment efficiency (EE) and loading capacity (LC)

The essential oil content of β-CD inclusion compounds was determined with the method that mentioned by Lin et.al with minor revision. To put in a nutshell, 0.1 g of each Citral@β-CD and Linalool@β-CD was dissolved in centrifuge tube with 20 mL of ethanol. The centrifuge tubes were tight lidded and the mixtures were sonicated for 30 min then subsequently placed at 4 °C overnight. Centrifuged the suspension at 5000 rpm for 20 min to remove the undissolved β-CD. Transferred the supernatant and to a volumetric flask and diluted to 100 mL. After an appropriate dilution, the absorbance of Citral@β-CD at 238 nm and Linalool@β-CD at 204 nm were measured using UV spectrophotometer (UV-2600 Shimadzu Co., Kyoto, Japan). The following equations calculated EE and LC of the inclusion complexes.

(3) $$\mathrm{EE}=\frac{\mathrm{Amount of encapsulated active compound}}{\mathrm{Initial amou}nt\;\mathrm{of active compound}}\times 100\%$$

(4) $$\mathrm{LC}=\frac{\mathrm{Amount of encapsulated active compound}}{\mathrm{Amount of particles produced}}\times 100\%$$

### 2.13 Scanning electron micrograph (SEM)

The dehydrated recrystallized β-CD, Citral@β-CD, Linalool@β-CD and CL@β-CD were gold coated and their micromorphology was observed by scanning electron microscopy (S-3400 N, Hitachi Ltd., Japan.) at an acceleration voltage of 5 kV. The captured magnification of all samples ranged from 1000× to 20000 × .

### 2.14 Particle size

Dynamic light scattering (DLS, Zetasizer Lab, Malvern) was used to determine particle size distribution. Both Citral@β-CD and Linalool@β-CD were prepared and a re-crystallized β-CD sample was also involved. The 10 mg of each particle sample were suspended in 1 mL of distilled water a and then transferred in 1 cm path length plastic cuvettes for particle size and polydispersity index (PDI) measurement (Song et al., 2024).

### 2.15 Application in cooked chicken

#### 2.15.1 Treatments

Fresh chicken breast was provided by a local supermarket. Whole fresh chicken breast was boiled in Milli-Q water for 10 min and was rinsed to remove the impurities. After that, cut the samples into 3 g cubes with disinfectant knife and randomly divided into 4 groups: (1) PBS; (2) Citral@β-CD; (3) Linalool@β-CD; (4) CL@β-CD. PBS-treated samples were used as controls. All chicken cubes were immersed in different prepared solutions and placed on sterile dishes. Adjusting *E. coli* suspension to ∼10^6^ CFU/mL and adding 50 μL on each the coated and control samples. All groups were stored at 4 °C and analyzed at day 0 and 3.

#### 2.15.2 Determination of total viable counts (TVC)

The bacterial count of chicken meats under different treatment was determined with the method introduced by Yang et al. (2023) that had minor modifications. Each sample (3 g) was mixed with 27 mL pre-sterile PBS in a centrifuge tube and homogenized for 1 min using an aseptic homogenizer (Shanghai HuXi Analysis Instrument Factory Co., Ltd., China). To figure out the bacterial amount of all treatment group, the homogeneous liquid sample were firstly subjected to a ten-fold gradient dilution. Next, 100 μL of diluted sample was spread evenly on NA plate and incubated at 37 °C for 24 h. TVC was finally measured by the tilting plate method.

#### 2.15.3 Total volatile basic nitrogen (TVB-N)

The TVB-N content of boiled chicken breast was analyzed by semimicro Kjeldahl method as described by Zhou et al. (2024). Specifically, 3 g of chicken meat sample were homogenized with 27 mL of PBS solution. Subsequently, 10 mL prepared liquid sample was mixed with 10 g/L magnesium oxide solution at the ratio of 2:1 and added to a Kjeldahl flask for distillation for 10 min. The distillate was absorbed by 10 mL of a mixture of 20 g/mL boric acid and methyl blue-methyl red and titrated with 0.01 mol/L HCl solution. The titration volume was then calculated according to the following equation and the results are expressed as mg/100 g meat.

(5) $$X=\frac{\left(V_{1}-V_{2}\right)\times C\times 14\times 30}{m\times 10}\times 100$$

where *X* represents the TVB-N content in chicken breast (mg/100 g); *V*_1_ and *V*_2_ represent the volume of HCl standard solution consumed by the experimental sample and the blank, *C* is the actual concentration (mol/L) of HCl used; and m represents the sample weight (g). Each sample was analyzed in triplicate, and the average was taken as the final TVB-N value.

#### 2.15.4 pH value

Chicken breast samples (3 g) were homogenized with 27 mL of PBS at 8000 rpm for 60 s. Tissue homogenate pH values were quantified with a calibrated digital pH analyzer (PHBJ-206, Leici, Shanghai, China). The pH meter was calibrated with pH standard buffers of 6.86 and 9.18 before each sampling days.

#### 2.15.5 Color parameters

The color value L* (lightness), a* (redness), and b* (yellowness) of chicken breast on the surface was measured using a spectrophotometer (CR-10 Plus, Konica Minolta, Optics, Inc.) during 4 °C storage. The spectrophotometer was calibrated using standardized whiteboards and chalkboards before the measurements.

### 2.16 Statistical analysis

To ensure reproducibility and robustness, all experiments were performed in triplicate. Data are expressed as mean values ± standard deviation (SD). Statistical significances among groups were analyzed using one-way ANOVA (GraphPad Prism 9.0), with statistical significance defined as *p* < 0.05. Intergroup comparisons are denoted as follows: * (*p* < 0.05), ** (*p* < 0.01), *** (*p* < 0.001), and **** (*p* < 0.0001).

## 3 Results and discussion

### 3.1 MIC and FIC

The MIC values of all tested essential oil compounds and their FIC values combined with linalool for *E. coli* are presented in Table 1. All compounds analyzed in this study exhibit antibacterial activity. Specifically, when used individually, the MIC values of eugenol, citral and linalool were determined to be 0.63 mg/mL, 0.75 mg/mL and 1.5 mg/mL, respectively. Notably, the checkerboard test results demonstrated that when the concentrations of eugenol, citral and linalool were 1/4 MIC, the FIC values of eugenol/linalool and citral/linalool combinations were both 0.5, indicating a significant synergistic antibacterial effect of these compound pairs against *E. coli*. In previous studies, the FIC index for the combination of citral and carvacrol against *C. sakazakii* CICC 21544 was 0.5 (Cao, Zhou, et al., 2021). For the combination of carvacrol and 1,8-cineole, the FIC index was determined to be 0.5 when tested against *S. aureus* (Honório et al., 2015). Similarly, the FIC indices for cinnamaldehyde combined with citronellal or nonanal vapors against *Aspergillus flavus* were reported as 0.5 and 0.25, respectively (Zhang et al., 2023). These findings are consistent with and provide support for the synergy results observed in our study. The combined antimicrobial effect of eugenol and linalool has been verified in previous studies on *L.* *monocytogenes, E. aerogenes, E. coli* and *P. aeruginosa* (Rehab & Zeinab, 2016). However, the synergistic effect of citral and linalool was first identified in the present study. Thus, citral/linalool combination was selected for subsequent analyses.

**Table 1:** MIC and FIC of citral and linalool against *E. coli*.

Antibacterial agents		Single MIC (mg/mL)		Combined MIC (mg/mL)		FIC	Combined effect
A	B	A	B	A	B		
Linalool	Carvacrol	1.5	0.16	0.38	0.08	0.75	additive
	Citronellal		2.50	0.75	0.63	0.75	additive
	Cinnamaldehyde		0.16	0.75	0.08	1	additive
	Citral		0.75	0.38	0.19	0.5	synergistic
	Thymol		0.16	0.75	0.04	0.75	additive
	Vanillin		1.25	0.38	1.25	1.25	indifferent
	Eugenol		0.63	0.38	0.16	0.5	synergistic

Synergistic (FIC index ≤0.5), additive (0.5 < FIC index ≤1), indifferent (1 < FIC index ≤4) or antagonism (FIC index >4).

To provide the evidence of the broad-spectrum synergistic antimicrobial activity of the citral and linalool combination, additional experiments were performed to evaluate its efficacy against *S. aureus*, *L. monocytogenes*, *Penicillium* spp., and *A. niger.* The MIC and FIC analyses revealed that citral-linalol combination exhibited a synergistic effect against *L. monocytogenes* and *A. niger*, with FIC indices of 0.375 for both strains (Table S1). In contrast, the effect against *S. aureus* and *Penicillium* spp. was additive (FIC = 1) (Table S1). These findings confirm that citral-linalool combination exhibits selective synergistic antimicrobial activity, the underlying mechanisms of which warrant further exploration.

### 3.2 Synergistic antibacterial effects in vitro

#### 3.2.1 Bacteria count

**Fig. 2:**
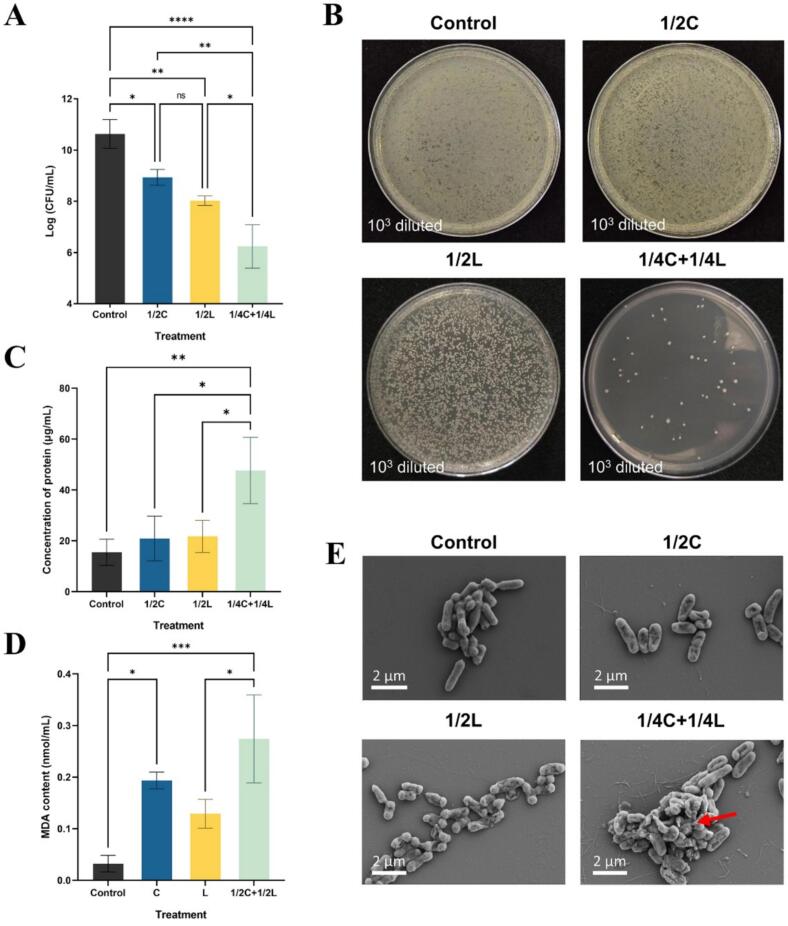
Inhibition effect of different treatments on *E. coli* growth after 24 h incubation at 37 °C: (B) photographs of nutrient broth (NB) media after a 1:10^3^ dilutions. (C) Extracellular protein content after 2 h incubation, (D) MDA content. (E) SEM image of *E. coli* treated with 1/2C, 1/2L, 1/4C + 1/4 L and control. Values are expressed as means (*n* = 3) ± SD (*p < 0.05; **p < 0.001, ****p < 0.0001). Abbreviations: 1/2C or 1/2 L means the treatments with 1/2 MIC of citral or 1/2 MIC of linalool, respectively. 1/4C + 1/4L means the combination treatment with 1/4 MIC of citral and 1/4 MIC of linalool.

The bacterial count results (Fig. 2A) demonstrate that after 24 h of incubation, the bacterial counts in the control group increased from an initial value of 5 Log CFU/mL to 10.63 Log CFU/mL. In contrast, treatment with citral (1/2 MIC) or linalool (1/2 MIC) individually resulted in final bacteria counts of 8.94 Log CFU/mL and 8.02 Log CFU/mL, showing inhibit rates of 15.90 % and 24.65 %, respectively, indicating their significant antibacterial effects. Notably, the combination of the two compounds of 1/4 MIC each resulted in a final bacterial count of 6.24 Log CFU/mL, which was significantly lower than that of all other groups. Meanwhile, the bacterial inhibition rate in the combined group reached 41.30 %, which was 2.60 and 1.68 times higher than those in the individual citral and linalool groups, respectively. Accordingly, Fig. 2B displays bacterial suspensions diluted at a ratio of 1:10^3^ and incubated on NA media for 24 h. Among all groups, the groups treated with a combination of 1/4 MIC of citral and 1/4 MIC of linalool exhibited significantly lower bacterial counts. These findings suggest that the combined treatment of citral and linalool achieves superior antibacterial efficiency compared to individual treatments. The inhibition of bacterial growth may be attributed to the biological effects of citral and linalool. Kang et al. (2022) demonstrated that citral could alter cellular morphology and intracellular soluble proteins, reduce cell membrane integrity and intracellular ATP concentration, induce cell membrane hyperpolarization, and elevate ROS levels of *Yersinia enterocolitica*. Gao et al. (2023) found that linalool treatment down-regulated biological processes related to ribosome assembly, biosynthetic and metabolic, protein-containing complexes, and peptides, as revealed by transcriptomic and proteomic analyses. These findings indicated that linalool strongly inhibits ribosomal assembly and protein synthesis in *E. coli*, leading to significant cell death.

#### 3.2.2 Intracellular protein leakage

Considering that citral has a strong absorption at the wavelength of 280 nm, the BCA protein assay was applied to avoid interference from essential oil components. After a co-incubation for 2 h, extracellular protein was detected in all samples. However, compare to control group (15.49 μg/mL), both individual treatment groups (1/2C and 1/2L) showed a minor increase of extracellular protein levels, with the concentrations of 20.89 μg/mL and 21.73 μg/mL, respectively (Fig. 2C). In addition, a rapid protein leakage reaching 47.64 μg/mL was found in the combined treatment group, which was significantly higher than that in the control group (*p* ≤ 0.01) and the individual groups (*p* ≤ 0.05). Proteins are indispensable biomacromolecules for bacterial growth, reproduction, and environmental adaptation, performing diverse vital functions within bacterial cells. During the growth of bacteria, a limited amount of specific proteins may be secreted out of the cell to play a role of intercellular signaling molecules or participate in metabolic processes. However, abnormal release of intracellular proteins is commonly linked to bacterial death and suggests cell membrane damage caused by antibacterial agents. The BCA test result demonstrated that both citral and linalool could induce intracellular protein leakage. Nevertheless, their single use had no difference in membrane breaking. By contrast, their combination resulted in a twofold increase in protein leakage concentration, proving that citral and linalool exerted a significant impact on the integrity of the bacterial cell membrane.

#### 3.2.3 MDA content

MDA is one of the end products of lipid peroxidation in living organisms, which is often used as the indicator of measuring the extent of oxidative damage to cells. As exhibited in Fig. 2D, only a trace amount of MDA was detected in the control group (0.03 nmol/mL). Compare with the control group, the MDA content in the group treated with citral at MIC for 1 h was 0.20 nmol/mL, indicating a significant induction of lipid peroxidation. In addition, the linalool MIC group showed a slight increase in MDA content to 0.12 nmol/mL. Meanwhile, the combined group (1/2C + 1/2L) presented the highest MDA level among all experiment groups, reaching 0.27 nmol/mL—approximately 1.35 and 2.25 times higher than that in the citral and linalool single-treatment groups, respectively, and even 9.00 times higher than that in the control group. The intense peroxidation effect induced by citral may be attribute to its unique chemical structure. Citral is an aldehyde monoterpene with strong oxidizing property due to the presence of an α, β-unsaturated aldehyde group, which brings it high chemical reactivity. Citral molecules can bind with the phospholipid bilayer closely and induce lipid oxidation in the cell membrane. Cao, Liu, et al. (2021) reported that ctiral upregulate the MDA content in *Vibrio parahaemolyticus* cultures with the increase of the concentration and the exposure time. Furthermore, He et al. (2023) proved that MDA contents, along with other ROS-related factors such as H_2_O_2_ and O_2_•^−^, increased after *Shigella sonnei* cultures were exposure to linalool. However, linalool, a monoterpene alcohol with a hydroxyl group, lacks the ability to readily accept electrons. Resulting in relatively weaker lipid peroxidation induction than citral due to differences in structure and properties.

#### 3.2.4 Morphology of *E. coli* cells

The morphological changes of *E. coli* exposed to various concentrations of citral, linalool and their combinations were analyzed using SEM. As shown in Fig. 2E, *E. coli* cells from the control group exhibited a rod-like shape, plump and the surface were smooth. Treatment with 1/2 MIC citral resulted in mild shrinkage of bacterial cell wall while the overall shape remained clear (Fig. 2E). In contrast, *E. coli* cells treated with linalool displayed wrinkling and slight collapse of their structure, yet the boundaries of their cell walls remained distinct (Fig. 2E). The image of the 1/4C + 1/4L group revealed severe cellular disruption, with more shriveled cells and adhesion between them (red arrow). However, the extent of cellular damage varied within this group. Some cells were less affected and retained their structural integrity. With the increasing concentrations of combined treatments, exposure to the citral/linalool mixture at 1/2 MIC+1/2 MIC induced serious cell deformations, making the shapes of the cells difficult to identify (Fig. 2E). Irregular surface textures and apparent disintegration of cell morphology was observed in this sample, with most of cells becoming shorter and cracked. It is obviously that the rupture of cell morphology is related to the concentration of the citral/linalool group. Similar to the protein leakage assay described above, when cells were exposed to citral or linalool at the same MIC ratio, the permeability of the cell wall and membrane was up-regulated, leading to the loss of cytosolic materials. Several studies have indicated that both citral and linalool have the ability to cause cell surface damage, thereby inducing bacterial inactivation and even death (Dai et al., 2023; Guo et al., 2021; Liu et al., 2020). Meanwhile, direct contact with the citral/linalool mixture also resulted in a concentration-dependent synergistic effect on the structural breakdown of *E. coli* cells. At the same absolute MIC, cell fracture was more significant: cells exhibited malformation and even developed holes. Gao et al. (2023) also demonstrated that linalool can affect the synthesis and modification of lipopolysaccharides, thereby damaging the outer membrane of *E. coli* cells and triggering cell envelope stress responses. Once the first line of bacteria self-defense broke down, the lipophilic citral was allowed to penetrate the cytoplasmic membrane, allowing a greater amount of the antibacterial agent to enter the cell interior. Consequently, further cell disruption occurred, followed by the rapid outflow of organelles and functional components.

#### 3.2.5 Biofilm formation

**Fig. 3:**
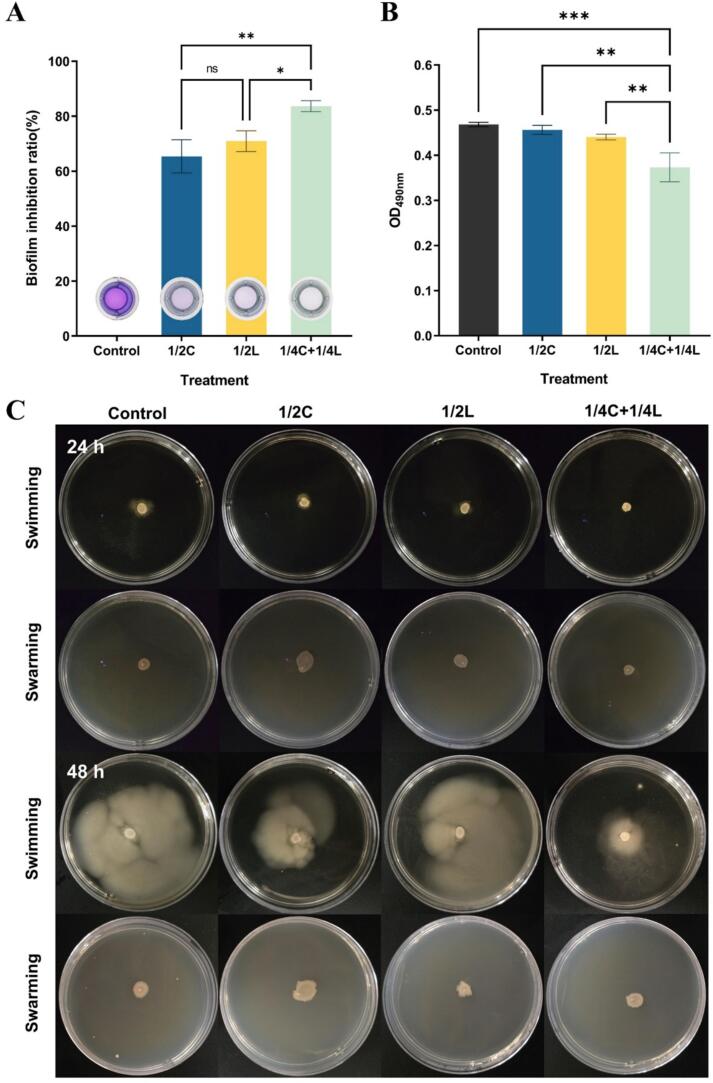
(A) Biofilm inhibition ratios after incubation for 48 h at 37 °C under different treatments. (B) Effect on EPS of *E. coli*. (C) Effects on the motility of *E. coli*.

The ability to control biofilm formation was observed in all groups studied (Fig. 3A). Specifically, the individual use of citral (1/2 MIC) or linalool (1/2 MIC) resulted in biofilm inhibition rates of 65.43 % and 70.95 % after 48 h of incubation, respectively, which corroborated previous findings (Cao, Zhou, et al., 2021; Pereira et al., 2018). When citral and linalool were applied in combination (1/4 MIC each), the biofilm inhibition rate reached 83.67 % after 48 h, representing an increase of 18.24 % and 12.12 % compared to the single use of cital and linalool, respectively. This finding verified their synergistic effect on inhibiting biofilm formation. There was previous research demonstrating that borneol and citral act synergistically to inhibit biofilms of both Gram-positive and Gram-negative foodborne bacteria, including *P. aeruginosa, E. coli, S. aureus,* and *L.* *monocytogenes* (Wang et al., 2025)*.* According to the bacteria count result presented above (Fig. 2A), the combination of citral and linalool led to a significant reduction in *E. coli* growth. Since biofilm formation relies on a self-produced polymeric matrix secreted by bacteria, a reduced bacterial population correlates with decreased secretion of metabolites, which in turn may lead to a reduction in biofilm formation.

#### 3.2.6 EPS content

In order to further explore the synergistic biofilm control mechanism of citral and linalool, EPS content after incubating for 48 h was analyzed. More precisely, co-incubation with individual citral and linalool (1/2 MIC) resulted in decreased EPS contents by 2.62 % and 6.00 %, respectively, compare to the control group (Fig. 3B). By contrast, a great reduction in EPS excretion during biofilm formation was recorded in the combined group (1/4C + 1/4L) at 20.33 % (*p* ≤ 0.001). The formation of the biofilm matrix by *E. coli* is significantly attributed to the production of EPS, which are crucial for biofilm structure and function. In turn, EPS provides a diffusion barrier against antibacterial agents and shields cells from unfavorable environmental conditions (Harimawan & Ting, 2016). Connecting with the biofilm formation assay earlier in this discussion, the synergistic group of citral and linalool effectively suppressed the growth of *E. coli*, along with a greater reduction in EPS. Correspondingly, the lower EPS content in the biofilm environment, the looser its structure becomes. Thus, its adhesive properties will be impaired, the elasticity of the biofilm will deteriorate, and the defense system for the bacteria will break down. Thereby, system imbalance is created, and bacteria in that environment undergoes escalating assaults from antibacterial agents.

#### 3.2.7 Swimming and swarming test

Likewise, flagella-mediated motility enables bacteria to possess a robust capability for colonizing host cells and adhering to surfaces, which is essential for the initial formation of biofilms. Additionally, motility also contributes to the escape of bacteria from mature biofilms to induce further infection and play a vital role in the virulence level of pathogenic bacteria (Chaban et al., 2015; Verstraeten et al., 2008). Swimming and swarming abilities were tested to investigate the combine effect of citral and linalool on bacterial motility in this study. It can be observed from Fig. 3C that, during the first 24 h, neither semisolid plates nor solid agar plates exhibited apparent bacterial colony spreading. Additionally, there was only a minor difference in colony diameter between the control and treatment groups in the swarming test. However, after 48 h of incubation, clear and well-developed bacterial halos were observed in the swimming test groups, whereas no significant differences were noted in the swarming test groups. Notably, when *E. coli* was exposed to 1/4C + 1/4L, a maximum decline in swimming motility with a faint and limited halo spread was observed compared to the control group. Individual administration of citral (1/2 MIC) had a less impact on swimming motility, while linalool (1/2 MIC) exhibited minimal effect on motility.

### 3.3 Physical properties of the prepared inclusion complexes

**Fig. 4:**
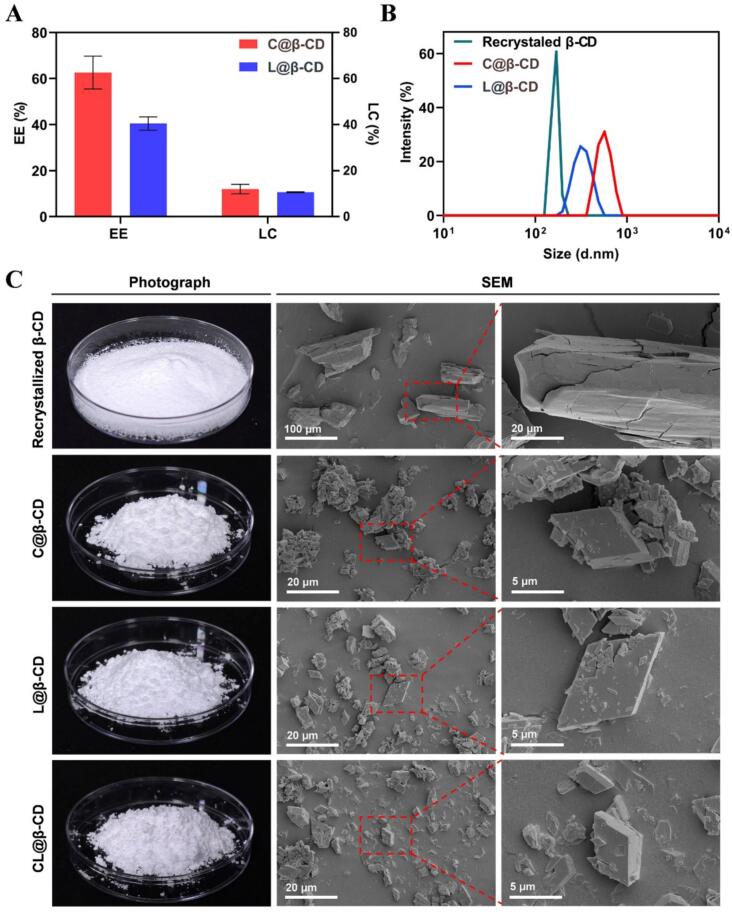
Characterization of β-CD inclusion complexes: (A) Entrapment efficiency (EE) and loading capacity (LC) of Citral@β-CD and Linalool@β-CD inclusion; (B) Particle size distribution curves of re-crystallized β-CD, Citral@β-CD and Linalool@β-CD; (C) Appearance photos and SEM images of β-CD inclusion complexes. Abbreviations: C@β-CD: Citral@β-CD, L@β-CD: Linalool@β-CD, CL@β-CD: Citral@β-CD + Linalool@β-CD.

The dried Citral@β-CD and Linalool@β-CD appeared as white fine powders with a dstinct aroma characteristic of the essential oils. The EE and LC of the Citral@β-CD inclusion complexes were 62.60 % and 11.95 %, respectively, while those for Linalool@β-CD were 40.47 % and 10.62 %, respectively (Fig. 4A). The preparation and applications of β-CD inclusion of essential oil components has been extensively studied over the past few decades. For example, Maswal and Dar (2014) reported that β-CD can be used as a good carrier of citral. López and Pascual-Villalobos (2010) synthesized a series of β-CD-monoterpenoids microcapsules, achieving maximum EE of 52 % with camphor, 34 % with geraniol, and 31 % with linalool. The EE and LC values can be affected by multiple factors such as the chemical properties of essential oil components, preparation methods, preparing temperature, mixing time and speed, the use of co-solvent or other additives, etc. (Kfoury et al., 2018), the results obtained in this study are considered reliable and valid.

SEM results of the β-CD complexes are presented in Fig. 4C. *Re*-crystallized β-CD preformed the large aggregations of irregular long prismatic crystals, whereas both Citral@β-CD and Linalool@β-CD composites demonstrated rhombic crystals. The surface of the inclusions was smooth, with no cracks, pores or fractures observed, suggesting that the compounds were well encapsulated in the inclusion complexes. The formation of such distinct morphologies indicates differences in the crystallization behavior and the interaction between the compounds and the β-CD.

The particle size distribution curves for different formulations are displayed in Fig. 4B. The particle diameters of pure β-CD, Citral@β-CD and Linalool@β-CD was 164.4 nm, 568.3 nm and 330.6 nm, respectively. The characteristic of the substance that package inside the β-CD shall may change its self-aggregation property and affect their crystal size. Besides, re-crystallized β-CD curve showed a polydispersity index (PDI) value of 1.00 while PDI of Citral@β-CD and Linalool@β-CD inclusion was 0.32 and 0.43, respectively. The PDI value is a key indicator of the uniformity of particle sizes in a suspension. A lower PDI value indicates a relatively more uniform particle size distribution, or a monodisperse system (Tao et al., 2014). Thus, the addition of essential oil compounds improved the dispersity of the complexes and facilitated a more even particle distribute within the system, suggesting a promising potential for practical applications. The interaction between β-CD cavities and essential oil molecules forms a stable complex through various non-covalent forces, including van der Waals forces, hydrogen bonds, dipole–dipole interactions, and London dispersion forces (Ravindran Maniam et al., 2022). These weak interactions enable controlled release of essential oil in response to specific condition changes, such as fluctuations in humidity, temperature, or mechanical stress, thereby facilitating a slow and sustained release profile.

### 3.4 Effects of extending the shelf life of cooked chicken

#### 3.4.1 TVC

**Fig. 5:**
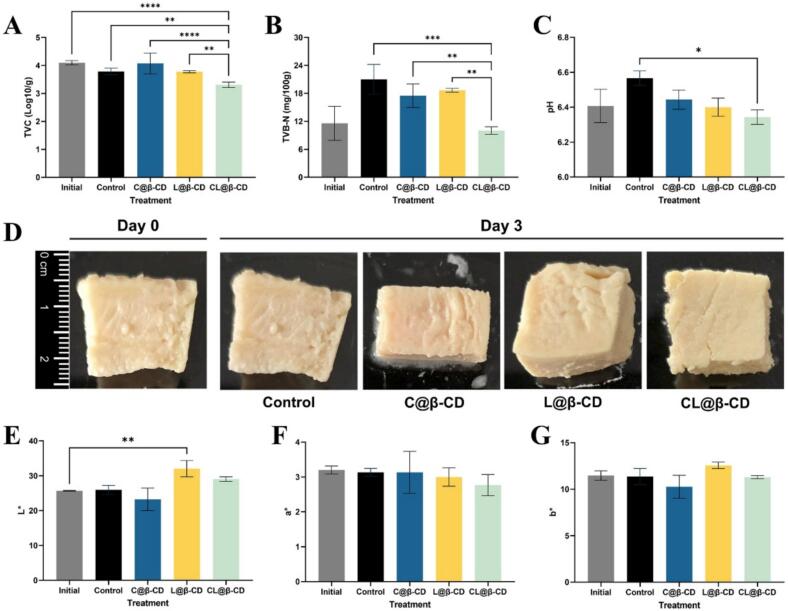
TVC (A), TVB-N (B), pH (C), photographs (D), and changes in color parameters: (E: L*, F: a*, and G: b*) of chicken samples during storage at 4 °C.

As shown in Fig. 5A, following the artificially inoculate of *E. coli*, the initial TVC of the chicken samples on day 0 was recorded as 4.10 Log CFU/g. By day 3, the TVC for the control group, Citral@β-CD, and Linalool@β-CD were maintained at 3.79, 4.07, and 3.78 Log CFU/g, respectively. In contrast, the CL@β-CD group exhibited a TVC of 3.31 Log CFU/g, which not only decreased from the initial value but also remarkably lower than those of all other groups. This finding is reasonable, as bacteria are inactive and their growth would be very slow or even suspend under 4 °C refrigerator conditions. Previous assessments have demonstrated that the combination of citral and linalool exhibits positive synergy when directly interacting with bacteria cells. Encapsulating essential oils within cyclodextrins reduces their volatility, thereby enabling long-term, low-concentration application on food surfaces (Kfoury et al., 2018). This approach extends the preservation period while preventing direct contact between the oils and food matrix, minimizing potential flavor alterations (Marques, 2010). When CL@β-CD was applied to cooked chicken meat, citral and linalool were slowly released, generating a synergistic antibacterial environment. Upon encountering *E. coli* on the chicken surface, linalool disrupts bacterial cell walls, thereby facilitating the entry of citral. This enables citral to more effectively induce lipid peroxidation and ROS generation, leading to MDA accumulation and membrane damage. As a result, essential oils can penetrate bacterial cells and cause further intracellular damage. The complementary mechanisms of these compounds enhance both bacterial inactivation and the inhibition of microbial growth. The decrease in TVC in CL@β-CD can be attributed to their strong synergistic antibacterial effects, demonstrating that the combined effect remains effective when applied in the form of β-CD complexes. In this study, citral and linalool were individually encapsulated and then physically blended. Although they do not contact with each other immediately, during storage, the gradual release process allows these volatile compounds to be delivered to the food surface and, perform synergistic antibacterial effects. Furthermore, the incorporation of these antibacterial compounds into β-CD enables sustained and long-term release, which helps inhibiting the development of TVC in boiled chicken meat.

#### 3.4.2 TVB-N

TVB-N compounds consist of primary, secondary, and tertiary amines in the form of volatile amines and toxic nitrogen compounds. Their contents are closely associated to the degradation of proteins and other nitrogenous substances produced by bacterial proliferation and often serve as a crucial biomarker for evaluating meat freshness (Bekhit et al., 2021). In this study, the initial TVB-N content of boiled chicken meat was 11.58 mg/100 g. After a three-day storage, the TVB-N contents increased in all groups except the CL@β-CD group. Specifically, the Citral@β-CD and Linalool@β-CD groups exhibited values of 17.50 and 18.67 mg/100 g, respectively, which were lower than that of the control group (21.00 mg/100 g) (Fig. 5B). Notably, the TVB-N content in the CL@β-CD treated group remained stable at 10.03 mg/100 g, showing no significant difference compared to the initial value. These findings indicated that the CL@β-CD treatment effectively inhibited the protein degradation in cooked chicken meat, which is consistent with the TVC results described above.

#### 3.4.3 pH value

The pH value also serves as a critical indicator of meat freshness. In this study, the initial pH value of the chicken meat was 6.40 (Fig. 5C). However, within day 3, the pH values of the control, Citral@β-CD, Linalool@β-CD and CL@β-CD groups were 6.57, 6.44, 6.40 and 6.34, respectively. Among them, only the CL@β-CD group presented a significant lower pH than the control (*p* ≤ 0.05). A low temperature and moisture environment can suppress the bacterial growth. However, the pH value of the control group started to raise on day 3, revealing that the proliferating *E. coli* degraded the protein and produced alkaline substances in the meat tissue. These results evidenced that the CL@β-CD coating have a superior ability in slowing down the spoilage of chicken breast and extending its shelf life.

#### 3.4.4 Colors

As shown in Fig. 5C, after a 3-day storage period, no significant differences were observed visually. However, the group coated with Linalool@β-CD showed a significant increase (*p* < 0.05) in L* values compare to the initial value (Fig. 5E). Furthermore, a* and b* value of the boiled chicken breast displayed in Fig. 5F-G did not find any significant deviation during the cold storage period (*p* > 0.05) in all groups. a* and b* stand for redness and yellowness, which closely connect with the content of myoglobin and metmyoglobin in meat (Ruiz-Hernández et al., 2023). During the process of high-temperature cooking, myoglobin in raw meat will be oxidized into metmyoglobin tend to stabilize (Ragucci et al., 2024). What is more, with low content of fat, the lipid oxidation products were correspondingly less, and the impact on the color of chicken was also limited. Base on the L*, a*, b* results above, the color varies between Linalool@β-CD and control was mainly attracted by lightness value. Combining with the TVC and TVB-N analyze result above, it can be seen that covering with CL@β-CD instead of Citral@β-CD or Linalool@β-CD was beneficial as it can not only maintain the appearance of boiled chicken breast but also providing remarkable antibacterial and preservation properties owning to the synergistic effect of citral and linalool.

## 4 Conclusion

In conclusion, this study firstly investigated the synergistic inhibitory effect of citral and linalool on *E. coli*. The combination significantly enhanced the efficacy of disrupting bacterial cell membranes, inducing protein leakage, promoting MDA accumulation, causing structural collapse, inhibiting biofilm formation, reducing EPS production, and impairing bacterial motility. Furthermore, the study also validated the effectiveness of combining their β-CD inclusion complexes in extending the shelf life of cooked chicken meat. The application of this composite agent as a coating effectively suppressed microbial growth, reduced TVB-N accumulation, and mitigated pH increase in the chicken meat. To extend the application of CL@β-CD, the inclusion can not only be applied to cooked meats but also to foods prone to *E. coli* contamination, such as raw diet vegetables, ready-to-eat dairy products, and fresh-cut fruits. Consequently, the findings of this study provide the food industry with an effective sustained-release antibacterial strategy based on the synergistic effects of natural plant-derived compounds.

## CRediT authorship contribution statement

**Wanying Zhu:** Writing – original draft, Visualization, Investigation, Data curation. **Yuhe Dong:** Investigation. **Tong Wu:** Investigation. **Haohan Jing:** Investigation. **Zikai Li:** Investigation. **Xi Yu:** Resources. **Ying Xiao:** Resources. **Tian Zhong:** Writing – review & editing, Supervision, Funding acquisition, Conceptualization.

## Declaration of competing interest

The authors declare that they have no known competing financial interests or personal relationships that could have appeared to influence the work reported in this paper.

## Data Availability

Data will be made available on request.

## Appendix A Supplementary data

Supplementary material Image 1
