# Supplementary material for: Beta-cyclodextrin inclusion complexes of citral and linalool inhibit Escherichia coli on cooked chicken: Focus on their synergistic antibacterial effects
Source: Food Chem X. 2025 Nov 4;32:103248. doi: 10.1016/j.fochx.2025.103248 (PMC12648581; doi:10.1016/j.fochx.2025.103248)
Supplement: Supplementary file 1 — Supplementary material [file mmc1.docx]

Table S1. The combined effect of citral and linalool against various microorganisms.

| Microorganisms | Single MIC (mg/mL) | | Combined MIC (mg/mL) | | FIC | Combined effect |
| --- | --- | --- | --- | --- | --- | --- |
|  | Citral | Linalool | Citral | Linalool |  |  |
| *Staphylococcus aureus* | 3.00 | 6.00 | 1.50 | 3.00 | 1.00 | additive |
| *Listeria monocytogenes* | 0.09 | 0.75 | 0.02 | 0.09 | 0.375 | synergistic |
| *Penicillium* spp. | 0.38 | 3.00 | 0.19 | 1.50 | 1.00 | additive |
| *Aspergillus niger* | 0.19 | 1.50 | 0.02 | 0.375 | 0.375 | synergistic |

Synergistic (FIC index ≤ 0.5), additive (0.5 < FIC index ≤ 1), indifferent (1 < FIC index ≤ 4) or antagonism (FIC index > 4).
